# Gastroesophageal reflux disease influences blood pressure components, lipid profile and cardiovascular diseases: Evidence from a Mendelian randomization study

**DOI:** 10.1515/jtim-2024-0017

**Published:** 2024-11-06

**Authors:** Qiang Wu, Changjing He, Wanzhong Huang, Chaoqun Song, Xin Hao, Qing Zeng, Dazhi Lan, Qiang Su

**Affiliations:** Department of Cardiology, Jiangbin Hospital of Guangxi Zhuang Autonomous Region, Guangxi Zhuang Autonomous Region, Guangxi China; Senior Department of Cardiology, the Sixth Medical Center, Chinese PLA General Hospital, Beijing, China; Journal of Geriatric Cardiology Editorial Office, Chinese PLA General Hospital, Beijing, China; Department of Pediatric Surgery, The Affiliated Hospital of Youjiang Medical University for Nationalities, Baise, China; Key Laboratory of Molecular Pathology for Hepatobiliary Diseases of Guangxi, Guangxi Zhuang Autonomous Region, Guangxi China; Department of Cardiology, the First Medical Center, Chinese PLA General Hospital, Beijing, China; Health Management Institute, the Second Medical Center, Chinese PLA General Hospital, Beijing, China; School of Public Health and Management, Guangxi University of Chinese Medicine, Guangxi Zhuang Autonomous Region, Guangxi China

**Keywords:** gastroesophageal reflux disease, blood pressure components, lipid profile, disease risk, Mendelian randomization

## Abstract

**Background:**

Gastroesophageal reflux disease (GERD) is a prevalent gastrointestinal disorder associated with a range of cardiovascular and metabolic complications. However, the relationship between GERD and blood pressure components, lipid profile, and cardiovascular diseases remains unclear.

**Methods:**

Leveraging genetic variants associated with GERD as instrumental variables, we performed this Mendelian randomization (MR) analyses. Blood pressure components, lipid profile parameters, as well as cardiovascular diseases were considered as outcomes. Furthermore, we conducted reverse MR analysis to explore the association of these factors with the risk of GERD.

**Results:**

Our MR analysis discovered a potential causal influence of GERD on blood pressure components, with genetically predicted GERD positively associated with systolic blood pressure (β = 0.053, *P* = 0.036), diastolic blood pressure (β = 0.100, *P* < 0.001), and mean arterial pressure (β = 0.106, *P* < 0.001). Additionally, genetically predicted GERD showed a significant impact on lipid profile, leading to increased genetically predicted levels of low-density lipoprotein (LDL) cholesterol (β = 0.093, *P* < 0.001), and triglycerides (β = 0.153, *P* < 0.001), while having a negative effect on high-density lipoprotein (HDL) cholesterol (β = -0.115, *P* = 0.002). Furthermore, our study indicated a noteworthy causal association between genetically predicted GERD and increased risk of myocardial infarction [odds ratio (OR) = 1.272, *P* = 0.019)] and hypertension (OR = 1.357, *P* < 0.001). No significant association was found between GERD and pulse pressure, total cholesterol, heart failure, and atrial fibrillation (*P* > 0.05). Reverse MR analysis indicates that blood pressure components, lipid profile, and cardiovascular diseases do not lead to an increased risk of GERD (all *P* > 0.05). Furthermore, mediation MR analysis reveals that LDL cholesterol (proportion mediated: 19.99%, 95% CI: 4.49% to 35.50%), HDL cholesterol (proportion mediated: 11.71%, 95% CI: 5.23% to 18.19%), and hypertension (proportion mediated: 35.09%, 95% CI: 24.66% to 45.53%) mediated the effect of GERD on myocardial infarction, while other factors did not participate in this pathway.

**Conclusions:**

This MR study provides evidence supporting a causal relationship between GERD and alterations in blood pressure components, lipid profile, and increased risk of cardiovascular diseases.

## Introduction

Gastroesophageal reflux disease (GERD),^[1,2,3]^ a common gastrointestinal disorder, has been traditionally associated with symptoms such as heartburn and regurgitation. However, emerging evidence suggests that the impact of GERD extends beyond the confines of the esophagus, with potential implications for cardiovascular and metabolic health.^[4,5,6]^ However, there remains controversy regarding the association between GERD and lipid profile and cardiovascular diseases. For instance, the study by Ha *et al*.^[6]^ demonstrated that GERD patients have higher triglyceride levels compared to non-GERD patients, while no difference was found in total cholesterol and high-density lipoprotein (HDL) cholesterol. On the contrary, Kallel *et al*.^[7]^ study found no significant difference in triglyceride between GERD patients and GERD-free patients. For cardiovascular disease, studies by Huang *et al*.^[8]^ and Maret-Ouda *et al*.^[9]^ elucidate the association between GERD and a high risk of atrial fibrillation. However, research by Bunch *et al*.^[10]^ suggests that GERD does not lead to a high risk of atrial fibrillation.

Furthermore, it is essential to emphasize that conclusions from previous research are subject to potential confounding factors, such as age, body mass index (BMI), medication usage, *etc*. To address the aforementioned challenge, we conducted a Mendelian randomization (MR) study, leveraging genetic instruments associated with GERD as instrumental variables. In addition, it is well-known that MR provides a robust method to assess causality by utilizing genetic variants as proxies for exposures, mitigating issues related to confounding factors that commonly present in observational studies.^[11,12]^ Thus, in this study, we perform an MR study aiming to elucidate the potential causal association between GERD and different outcomes, specifically focusing on blood pressure components [systolic blood pressure (SBP), diastolic blood pressure (DBP), pulse pressure (PP), and mean arterial pressure (MAP)], lipid profile parameters (low-density lipoprotein [LDL] cholesterol, HDL cholesterol, triglycerides, and total cholesterol), and disease risks including myocardial infarction, heart failure, atrial fibrillation, and hypertension.

## Methods

### Study design

**Figure 1:**
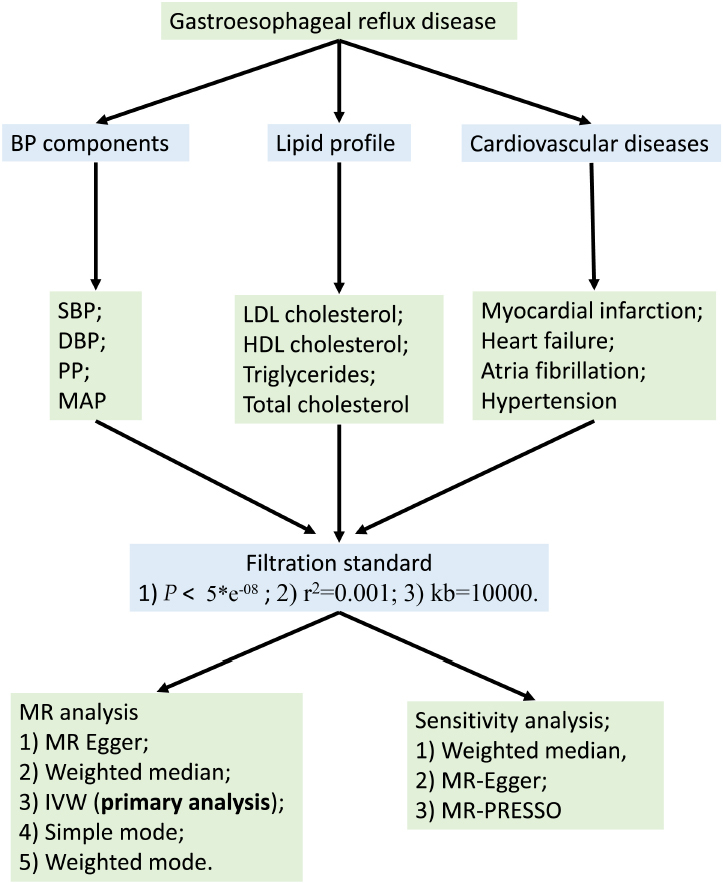
The flowchart of this Mendelian randomization study. BP components: blood pressure components; SBP: systolic blood pressure; DBP: diastolic blood pressure; PP: pulse pressure; MAP: mean arterial pressure; LDL cholesterol: low-density lipoprotein cholesterol; HDL cholesterol: high-density lipoprotein cholesterol; MR: Mendelian randomization; IVW: inverse variance weighted.

We conducted a MR analysis utilizing large summary statistics from genome-wide association studies (GWAS) to investigate the association between GERD and blood pressure components, lipid profile, and disease risk. Furthermore, we conducted reverse MR analysis to explore the association of these factors with the risk of GERD. MR employs single nucleotide polymorphisms (SNPs) as instrumental variables to explore the causal impact of exposure on outcome. This method is robust against confounding factors since SNPs are randomly distributed during meiosis following Mendel’s laws. The rationality of the MR analysis relies on three important assumptions:^[11,13]^ (1) a robust association between SNPs and the exposure; (2) the relationship between exposure and outcome remains unaffected by other confounding factors; and (3) SNPs associated with exposure exclusively influence outcomes through the exposure and are not influenced by alternative pathways. In this MR study, GERD serves as the exposure, while blood pressure components, lipid profile, and disease risk serve as outcomes. The blood pressure components encompass SBP, DBP, PP and MAP. The lipid profile includes LDL cholesterol, HDL cholesterol, triglycerides, and total cholesterol. Disease risk comprises myocardial infarction, heart failure, atrial fibrillation, and hypertension. The flowchart of this MR study is shown in Figure 1. The analytical process followed the guidelines outlined in the STROBE-MR guidelines.^[14]^

### Ethical consideration

This MR study adhered to ethical standards in the utilization of genetic and phenotypic data. The summary-level data used were attained from publicly website, and ethical approval was not required as the data were de-identified and did not involve direct contact with study participants. The study design prioritized privacy and confidentiality, ensuring compliance with ethical guidelines governing the responsible use of genetic information. Additionally, transparency and adherence to established research ethics principles were maintained throughout the analysis and reporting process.

### Data source

#### Data source for exposure

The large-scale GWAS data for GERD were derived from a study conducted by Ong *et al*.^[15]^ In Ong *et al*.’s study,^[15]^ a cohort of 602,604 participants of European descent was recruited, comprising 129,080 individuals diagnosed with GERD and 473,524 controls. Within this study, thorough identification and examination of 2,320,781 SNPs were conducted to investigate their potential associations with GERD.

#### Data source for different outcomes

The data for blood pressure components were sourced from two distinct studies. Information on SBP and DBP was extracted from the research conducted by Mbatchou *et al*.^[16]^ which included over 380,000 individuals of European ancestry. For SBP, details were obtained from 385,798 individuals, encompassing 10,783,907 SNPs, while for DBP, data were sourced from 385,801 individuals, involving 10,783,908 SNPs. PP and MAP data were drawn from the study conducted by Sakaue *et al*.^[17]^ which recruited over 360,000 individuals of European descent. Specifically, PP data were derived from 360,863 individuals, covering 19,047,322 SNPs, and MAP data were sourced from 360,863 individuals, involving 19,053,944 SNPs.

The data for the lipid profile came from four different studies. LDL cholesterol information was taken from the study led by Klimentidis *et al*.^[18]^ which had 431,167 participants of European descent and involved 16,293,344 SNPs. HDL cholesterol details were sourced from the research conducted by Mbatchou *et al*.^[16]^ which included 357,810 participants of European descent and had 10,783,660 SNPs. Triglycerides data were extracted from the study by Richardson *et al*.^[19]^ which enrolled 441,016 participants of European descent and included 12,321,875 SNPs. Total cholesterol data were obtained from the study conducted by Sakaue *et al*.^[17]^ involving 344,278 participants of European descent and encompassing 19,043,498 SNPs.

The data on cardiovascular diseases also come from four distinct studies. Myocardial infarction data were extracted from the study led by Sakaue *et al*.^[17]^ involving 461,823 participants of European descent. In this study, 20,917 individuals were diagnosed with myocardial infarction, while 440,906 were non-myocardial infarction cases, covering a total of 24,172,914 SNPs. Heart failure data were gathered from the research conducted by Shah *et al*.^[20]^ which included 977,323 participants of European ancestry. Among them, 47,309 individuals were diagnosed with heart failure, and 930,014 were non-heart failure cases, with a total of 7,773,021 SNPs. Atrial fibrillation data were acquired from the study by Nielsen *et al*.^[21]^ comprising 1,030,836 participants of European descent. In this cohort, 60,620 individuals were diagnosed with atrial fibrillation, and 970,216 were non-atrial fibrillation cases, involving a total of 33,519,037 SNPs. Hypertension GWAS data originated from the FinnGen dataset (ID number: finn-b-I9_HYPTENSESS_EXNONE), with 218,792 participants of European descent. Within this dataset, 42,857 individuals were diagnosed with hypertension, and 175,935 were non-hypertension cases, incorporating a total of 16,380,466 SNPs.

### Selection of SNPs

The procedure of SNPs selection was carried out utilizing the aforementioned GWAS databases, adhering to the three crucial assumptions of MR. To mitigate potential issues associated with linkage disequilibrium, a rigorous clustering procedure was performed, using clustering windows with an r^2^ = 0.001 and a kb = 10000. Furthermore, a threshold of *P* < 5*e^-08^ was employed to detect SNPs drastically associated with GERD. Additionally, an examination for potential confounding factors was conducted on the identified SNPs. Confounding factors encompassed age, gender, BMI, obesity, smoking, alcohol consumption, waist circumference, hip circumference, waist-hip ratio, depression, anxiety, HbA1c levels, blood glucose, tumors, diabetes, stress, obstructive sleep apnea, chronic kidney disease, multiple sclerosis, rheumatoid arthritis, potential diseases, and medication usage. SNPs associated with these confounding factors will be excluded from this MR study. The association between SNPs and confounding factors was assessed using the online platform PhenoScanner^[22]^ (http://www.phenoscanner.medschl.cam.ac.uk/), GWAS Catalog (https://www.ebi.ac.uk/gwas/), IEU OpenGWAS project (https://gwas.mrcieu.ac.uk/). Additionally, to exclude weak instrumental variables, the calculation of the F-statistic was performed. Instrumental variables that yielded an F-statistic of less than 10 were deemed weak and thus excluded from this study. Following previous literature,^[23,24]^ the formula for calculating the F-statistic is: F = (beta/se)^2^.

### Statistical analysis

In this study, we initially employed five different methods, including MR Egger, Weighted median, Inverse variance weighted (IVW), Simple mode, and Weighted mode, to explore the potential causal relationship between GERD and various outcomes. In addition, we utilized reverse MR analysis to investigate the effects of blood pressure components, lipid profile, and cardiovascular diseases on GERD. Among these, the IVW method was considered the primary analysis,^[25,26]^ while the other four methods were regarded as secondary analyses. In addition, we calculated Q statistics to assess the heterogeneity among the included instrumental variables, where a *P*-value of less than 0.05 indicated the presence of significant heterogeneity. In our study analysis, we employed random-effects IVW analysis when encountering significant heterogeneity (*P* < 0.05), and fixed-effects IVW analysis was used in cases where significant heterogeneity was absent (*P* > 0.05). For the sensitivity analysis, we applied three different methods, including Weighted median, MR-Egger, and MR-PRESSO,^[27]^ to assess the consistency of associations and address potential horizontal pleiotropy. We employed the Weighted median method^[28]^ to evaluate potential biases deriving from invalid instruments. If over 50% of the weight in the meta-analysis is based on valid SNPs, this method yields dependable estimates. Furthermore, MR-Egger^[29]^ was utilized to detect any potential directional pleiotropy. The p-value calculated in the MR Egger analysis is utilized to evaluate the presence of horizontal pleiotropy. A *P*-value < 0.05 indicates significant horizontal pleiotropy, while a *P*-value > 0.05 suggests the absence of significant horizontal pleiotropy. Additionally, in this study, MR-PRESSO analysis was also conducted to detect and address potential outlier SNPs. SNPs identified as outliers through this process will be removed from the study.

### Univariable and multivariable MR analysis

In this analysis, we considered cardiovascular diseases significantly associated with GERD (*P* < 0.05) as the outcomes, with blood pressure components and lipid profile significantly associated with GERD (*P* < 0.05) as exposures. Initially, we conducted univariable MR analysis to explore the effects of blood pressure components and lipid profile on cardiovascular diseases. Blood pressure components and lipid profile that exhibited significant associations with cardiovascular diseases were subsequently included in the multivariable MR analysis to further estimate the independent effects of each exposure on cardiovascular diseases.

### Mediation MR analysis

In the mediation MR analysis, we employed a two-step MR approach to investigate whether blood pressure components and lipid profile mediate the effect of GERD on cardiovascular diseases. In this process, GERD was considered as the exposure, while the blood pressure components and lipid profile significantly associated with cardiovascular diseases in the multivariable MR analysis were considered as mediators, with cardiovascular diseases as the outcome. The effect of GERD on blood pressure components and lipid profile was denoted as β1, and the effect of blood pressure components and lipid profile on cardiovascular diseases was denoted as β2. The total effect of GERD on cardiovascular diseases was denoted as β (total). The proportion of mediation by each blood pressure component in the association between GERD and cardiovascular diseases was calculated as β1 * β2 / β (total). The 95% confidence interval (CI) for the mediation effect was computed using the delta method.^[30]^

Within this study, results were depicted using β coefficients and 95% CI for continuous variables, while odds ratios (OR) and their corresponding 95% CI were employed for categorical variables. The data analysis for this MR investigation was carried out using RStudio software (version 4.2.2) along with the Mendelian Randomization, MRPRESSO, and TwoSampleMR packages. A *P*-value below 0.05 indicates significant statistical differences.

## Results

Several large GWAS datasets used in this MR study were obtained from previous studies, all involving European populations. The populations included in different studies all had sample sizes exceeding 200,000 individuals, with the smallest sample size being 2l8,792 and the largest being l,030,836. In summary, characteristics of the GWAS datasets used in this MR study are provided in Table 1.

**Table 1:** Characteristics of the GWAS datasets used in this mendelian randomization study

Type	PMID/ID	Journal	Population	Year	Sample size (case/control)	Number of SNPs
Gastroesophageal reflux disease	34187846	Gut	European	2021	602,604 (129,080/ 473,524)	2,320,781
Systolic blood pressure	34017140	Nat Genet	European	2021	385,798 (-/-)	10,783,907
Diastolic blood pressure	34017140	Nat Genet	European	2021	385,801 (-/-)	10,783,908
Pulse pressure	34594039	Nat Genet	European	2021	360,863 (-/-)	19,047,322
Mean arterial pressure	34594039	Nat Genet	European	2021	360,863 (-/-)	19,053,944
LDL cholesterol	32493714	Diabetes	European	2020	431,167 (-/-)	16,293,344
HDL cholesterol	34017140	Nat Genet	European	2021	357,810 (-/-)	10,783,660
Triglycerides	32203549	PLoS Med	European	2020	441,016 (-/-)	12,321,875
Total cholesterol	34594039	Nat Genet	European	2021	344,278 (-/-)	19,043,498
Myocardial infarction	34594039	Nat Genet	European	2021	461,823 (20,917/440,906)	24,172,914
Heart failure	31919418	Nat Commun	European	2020	977,323 (47,309/930,014)	7,773,021
Atrial fibrillation	30061737	Nat Genet	European	2018	1,030,836 (60,620/970,216)	33,519,037
Hypertension	finn-b-I9_HYPTENSESS_EXNONE	-	European	2021	218,792 (42,857/175,935)	16,380,466

GWAS: genome-wide association studies; SNPs: single nucleotide polymorphisms; LDL cholesterol: low-density lipoprotein cholesterol; HDL cholesterol: high-density lipoprotein cholesterol.

### Causal relationship between GERD and blood pressure components

**Figure 2:**
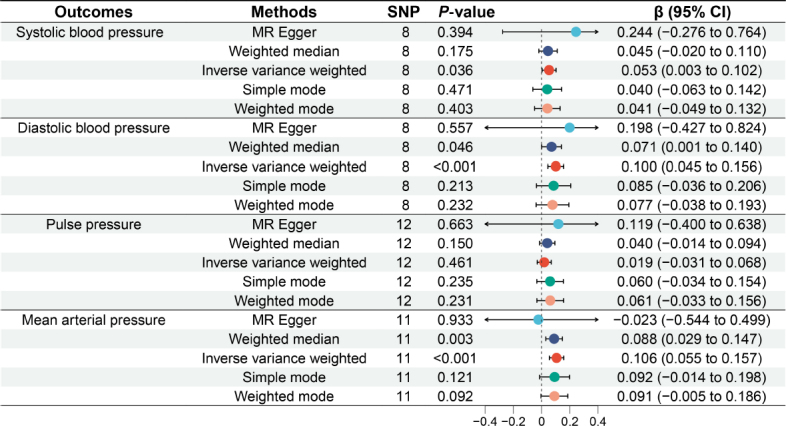
Causal relationship between gastroesophageal reflux disease and blood pressure components. MR: Mendelian randomization; SNP: single nucleotide polymorphism; CI: confidence intervals.

In Figure 2, we explore the causal links between GERD and blood pressure components. In the final analysis, we identified a total of 8 SNPs associated with SBP, 8 SNPs with DBP, l2 SNPs with PP, and ll SNPs with MAP. Subsequent analyses using the IVW method revealed a significant causal relationship between genetically predicted GERD and SBP, DBP, and MAP. Genetically predicted GERD led to a significant increase in SBP (β = 0.053, 95% CI: 0.003 to 0.102, *P* = 0.036), DBP (β = 0.100, 95% CI: 0.045 to 0.156, *P* < 0.001), and MAP (β = 0.106, 95% CI: 0.055 to 0.157, *P* < 0.001). However, no significant association was observed between genetically predicted GERD and PP (β = 0.019, 95% CI: -0.031 to 0.068, *P* = 0.461), regardless of the method used (MR Egger, Weighted Median, IVW, Simple Mode, Weighted Mode, *P* > 0.05).

### Causal relationship between GERD and lipid profile

**Figure 3:**
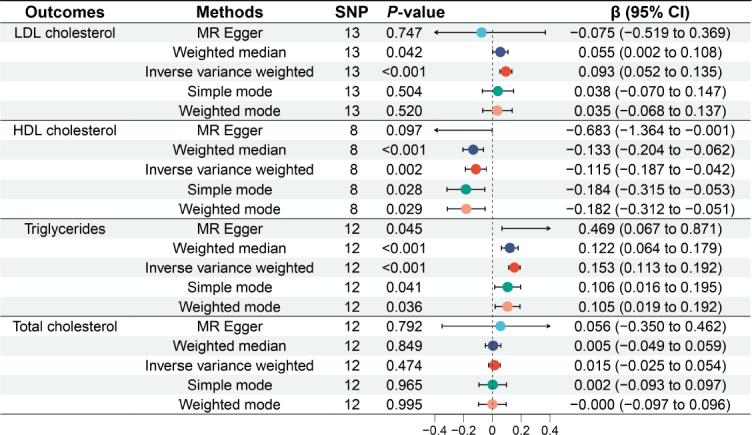
Causal relationship between gastroesophageal reflux disease and lipid profile. LDL cholesterol: low-density lipoprotein cholesterol; HDL cholestero high-density lipoprotein cholesterol; MR: Mendelian randomization; SNP: single nucleotide polymorphism; CI: confidence intervals.

Figure 3 illustrates the causal relationships between GERD and the lipid profile. A total of l3 SNPs were identified to be associated with LDL cholesterol, 8 SNPs with HDL cholesterol, 12 SNPs with triglycerides, and 12 SNPs with total cholesterol. Analyses using the IVW method indicated a significant causal relationship between genetically predicted GERD and LDL cholesterol, HDL cholesterol, and triglycerides. Genetically predicted GERD resulted in a significant increase in LDL cholesterol (β = 0.093, 95% CI: 0.052 to 0.135, *P* < 0.001) and triglycerides (β = 0.153, 95% CI: 0.113 to 0.192, *P* < 0.001), while causing a significant decrease in HDL cholesterol (β = -0.115, 95% CI: -0.187 to -0.042, *P* = 0.002). However, no significant association was found between genetically predicted GERD and total cholesterol (β = 0.015, 95% CI: -0.025 to 0.054, *P* = 0.474), regardless of the method used (*P* > 0.05 for each method).

### Causal relationship between GERD and risk of cardiovascular diseases

**Figure 4:**
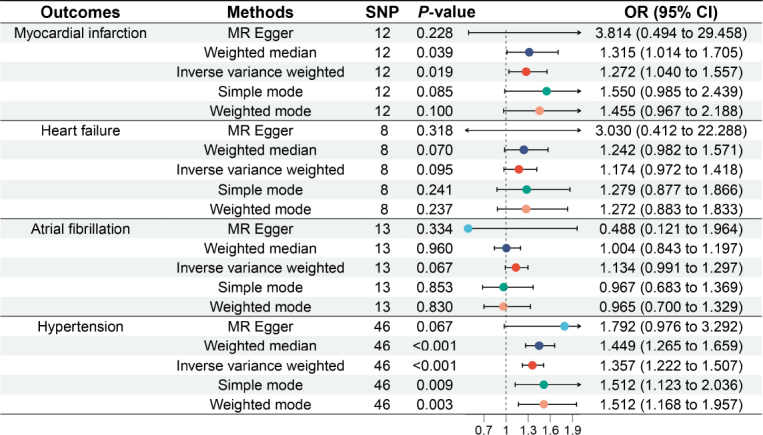
Causal relationship between gastroesophageal reflux disease and cardiovascular diseases. OR: odds ratio; MR: Mendelian randomization; SNP: single nucleotide polymorphism; CI: confidence intervals.

Figure 4 outlines the relationships between GERD and disease risk. A total of 12 SNPs were associated with myocardial infarction, 8 SNPs with heart failure, 13 SNPs with atrial fibrillation, and 46 SNPs with hypertension. IVW analyses revealed a significant causal link between genetically predicted GERD and myocardial infarction, as well as hypertension. Genetically predicted GERD significantly increased the risk of myocardial infarction (OR = 1.272, 95% CI: 1.040 to 1.557, *P* = 0.019) and hypertension (OR = 1.357, 95% CI: 1.222 to 1.507, *P* < 0.001). Nevertheless, no significant association was detected between genetically predicted GERD and heart failure (OR = 1.174, 95% CI: 0.972 to 1.418, *P* = 0.095) or atrial fibrillation (OR = 1.134, 95% CI: 0.991 to 1.297, *P* = 0.067), suggesting that the presence of genetically predicted GERD does not drastically increase the risk of heart failure and atrial fibrillation.

### Heterogeneity testing

We used two methods includin MR E er and IVW to evaluate the heterogeneity in the association between the exposure (GERD) and outcomes (SBP, DBP, PP, MAP, LDL cholesterol, HDL cholesterol, triglycerides, total cholesterol, myocardial infarction, heart failure, atrial fibrillation, hypertension). Detailed results are described in Table 2. Irrespective of the method used, whether MR Egger or IVW, we found no considerable heterogeneity in the relationship between GERD and SBP, DBP, MAP, LDL cholesterol, triglycerides, total cholesterol, myocardial infarction, heart failure, atrial fibrillation and hypertension (*P* > 0.05 for both methods). Additionally, MR Egger detected noteworthy heterogeneity in the relationship between GERD and PP (*P* = 0.036), while IVW suggested the relationship to be marginally significant (*P* = 0.050). Conversely, IVW detected considerable heterogeneity in the relationship between GERD and HDL cholesterol (*P* = 0.021), whereas MR Egger did not detect this significant heterogeneity (*P* = 0.077).

**Table 2:** Heterogeneity test between gastroesophageal reflux disease and different outcomes

Exposure	Outcomes	Methods	Q statistics	*P*-value
GRD	SBP	MR Egger	5.851	0.440
GRD	SBP	Inverse variance weighted	6.373	0.497
GRD	DBP	MR Egger	7.747	0.257
GRD	DBP	Inverse variance weighted	7.870	0.344
GRD	Pulse pressure	MR Egger	19.379	0.036
GRD	Pulse pressure	Inverse variance weighted	19.659	0.050
GRD	MAP	MR Egger	15.044	0.090
GRD	MAP	Inverse variance weighted	15.440	0.117
GRD	LDL cholesterol	MR Egger	15.145	0.176
GRD	LDL cholesterol	Inverse variance weighted	15.914	0.195
GRD	HDL cholesterol	MR Egger	11.399	0.077
GRD	HDL cholesterol	Inverse variance weighted	16.515	0.021
GRD	Triglycerides	MR Egger	8.762	0.555
GRD	Triglycerides	Inverse variance weighted	11.164	0.430
GRD	Total cholesterol	MR Egger	9.415	0.493
GRD	Total cholesterol	Inverse variance weighted	9.456	0.580
GRD	Myocardial infarction	MR Egger	12.240	0.269
GRD	Myocardial infarction	Inverse variance weighted	13.610	0.255
GRD	Heart failure	MR Egger	3.918	0.688
GRD	Heart failure	Inverse variance weighted	4.793	0.685
GRD	Atrial fibrillation	MR Egger	12.268	0.344
GRD	Atrial fibrillation	Inverse variance weighted	13.854	0.310
GRD	Hypertension	MR Egger	58.765	0.067
GRD	Hypertension	Inverse variance weighted	59.871	0.068

GRD: gastroesophageal reflux disease; MR: Mendelian randomization; SBP: systolic blood pressure; DBP: diastolic blood pressure; MAP: mean arterial pressure; LDL cholesterol: low-density lipoprotein cholesterol; HDL cholesterol: high-density lipoprotein cholesterol; SNPs: single nucleotide polymorphisms; CI: confident interval.

### Assessment of pleiotropy

To further investigate whether the relationship between GERD and SBP, DBP, PP, MAP, LDL cholesterol, HDL cholesterol, triglycerides, total cholesterol, myocardial infarction, heart failure, atrial fibrillation, and hypertension is influenced by other potential factors, we performed MR Egger regression to assess the presence of pleiotropy. The results displayed that each Egger intercept value was negligible (range from -0.034 to 0.026), with each *P*-value > 0.05, suggesting that the relationship between GERD and different outcomes is not affected by pleiotropy. Detailed results are provided in Table 3. Therefore, it can be concluded that the relationships between GERD and SBP, DBP, PP, MAP, LDL cholesterol, HDL cholesterol, triglycerides, total cholesterol, myocardial infarction, heart failure, atrial fibrillation, and hypertension are robust and not influenced by traditional confounding factors or potential confounders.

**Table 3:** Assessment of pleiotropy between gastroesophageal reflux disease and different outcomes

Exposure	Outcomes	Egger intercept	Se	*P*-value
Gastroesophageal reflux disease	Systolic blood pressure	-0.006	0.008	0.497
Gastroesophageal reflux disease	Diastolic blood pressure	-0.003	0.010	0.768
Gastroesophageal reflux disease	Pulse pressure	-0.003	0.008	0.712
Gastroesophageal reflux disease	Mean arterial pressure	0.004	0.008	0.638
Gastroesophageal reflux disease	Low density lipoprotein cholesterol	0.005	0.007	0.470
Gastroesophageal reflux disease	High density lipoprotein cholesterol	0.018	0.011	0.152
Gastroesophageal reflux disease	Triglycerides	-0.010	0.006	0.152
Gastroesophageal reflux disease	Total cholesterol	-0.001	0.006	0.844
Gastroesophageal reflux disease	Myocardial infarction	-0.034	0.032	0.315
Gastroesophageal reflux disease	Heart failure	-0.029	0.031	0.386
Gastroesophageal reflux disease	Atrial fibrillation	0.026	0.022	0.258
Gastroesophageal reflux disease	Hypertension	-0.009	0.010	0.367

### Reverse MR analysis

**Figure 5:**
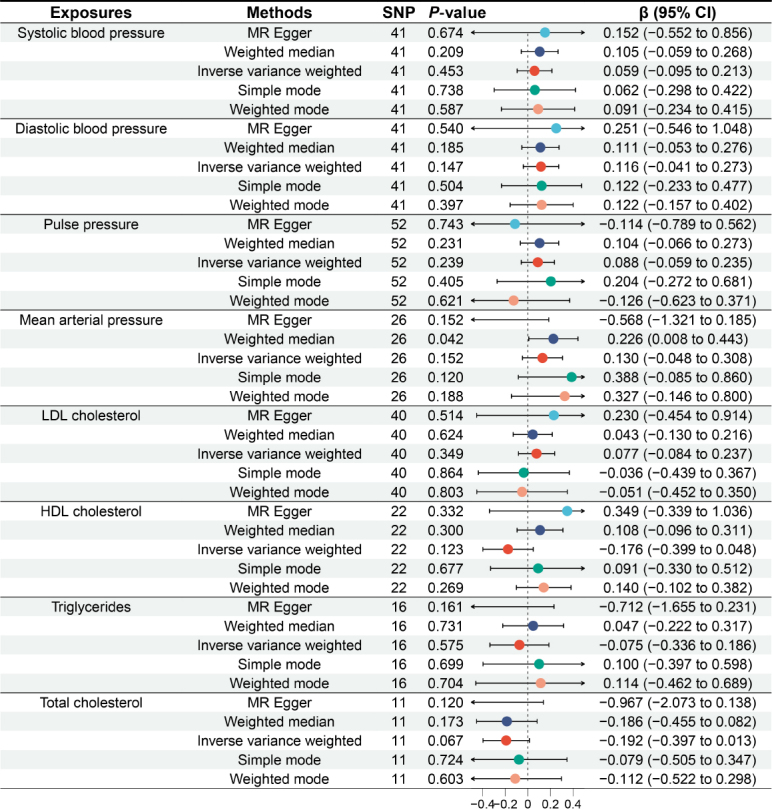
Causal relationship between blood pressure components, lipid profile and gastroesophageal reflux disease. MR: Mendelian randomization; SNP: single nucleotide polymorphism; CI: confidence intervals.

**Figure 6:**
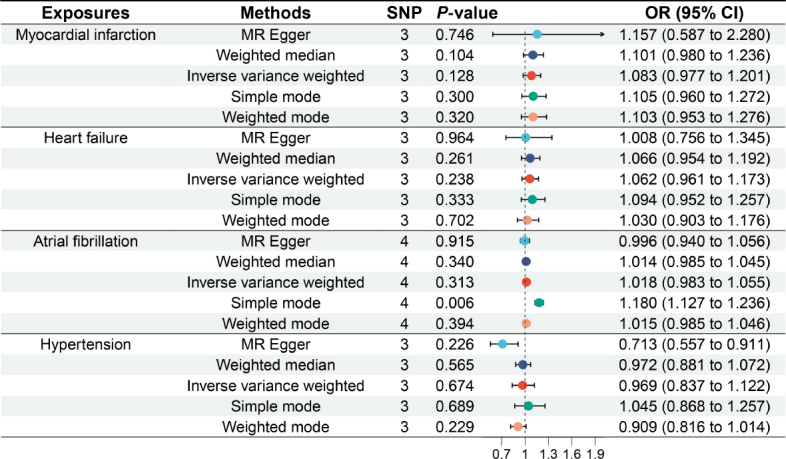
Causal relationship between cardiovascular diseases and gastroesophageal reflux disease. MR: Mendelian randomization; SNP: single nucleotide polymorphism; OR: odds ratio; CI: confidence intervals.

We further conducted a reverse MR analysis, using blood pressure components, lipid profile, and cardiovascular diseases as exposures, and GERD as the outcome. The results of the reverse MR analysis indicate that there is no significant (all *P* > 0.05) genetic association between blood pressure components, lipid profile, and cardiovascular diseases and the risk of GERD (Figure 5 and Figure 6).

### Univariable and multivariable MR analysis

We initially considered myocardial infarction as the outcome and other factors significantly associated with GERD as exposures (*P* < 0.05), using univariable and multivariable MR analysis to explore the relationship between myocardial infarction and these factors. Univariable MR analysis revealed that SBP (OR = 1.639, 95% CI: 1.355 to 1.983, *P* < 0.001), DBP (OR = 1.627, 95% CI: 1.399 to 1.893, *P* < 0.001), MAP (OR = 2.242, 95% CI: 1.805 to 2.785, *P* < 0.001), LDL cholesterol (OR = 1.678, 95% CI: 1.457 to 1.932, *P* < 0.001), HDL cholesterol (OR = 0.782, 95% CI: 0.694 to 0.882, *P* < 0.001), triglycerides (OR = 1.346, 95% CI: 1.094 to 1.658, *P* = 0.005), and hypertension (OR = 1.319, 95% CI: 1.152 to 1.509, *P* < 0.001) are associated with the risk of myocardial infarction. Detailed results are shown in Table 4. In the multivariable analysis, we found that LDL cholesterol (β = 1.748, 95% CI: 1.598 to 1.913, *P* < 0.001) and hypertension (OR = 1.355, 95% CI: 1.189 to 1.543, *P* < 0.001) are associated with an increased risk of myocardial infarction, while HDL cholesterol (OR = 0.807, 95% CI: 0.721 to 0.904, *P* < 0.001) is associated with a decreased risk of myocardial infarction (Table 5). We further explored the effects of LDL cholesterol, HDL cholesterol, triglycerides, and myocardial infarction on hypertension and found that LDL cholesterol (OR = 1.066, 95% CI: 0.924 to 1.229, *P* = 0.064), HDL cholesterol (OR = 0.900, 95% CI: 0.805 to 1.006, *P* = 0.062), triglycerides (OR = 1.170, 95% CI: 0.973 to 1.408, *P* = 0.096), and myocardial infarction (OR = 1.094, 95% CI: 0.994 to 1.204, *P* = 0.065) are not significantly associated with hypertension.

**Table 4:** Results of univariable analysis associated with myocardial infarction

Exposures	Methods	Number of SNP	OR	95% CI	*P*-value
Systolic blood pressure	MR Egger	133	1.655	0.714 to 3.837	0.243
Systolic blood pressure	Weighted median	133	1.633	1.332 to 2.002	<0.001
Systolic blood pressure	IVW	133	1.639	1.355 to 1.983	<0.001
Systolic blood pressure	Simple mode	133	1.259	0.739 to 2.146	0.398
Systolic blood pressure	Weighted mode	133	1.618	0.943 to 2.777	0.083
Diastolic blood pressure	MR Egger	118	1.284	0.683 to 2.416	0.439
Diastolic blood pressure	Weighted median	118	1.566	1.290 to 1.902	<0.001
Diastolic blood pressure	IVW	118	1.627	1.399 to 1.893	<0.001
Diastolic blood pressure	Simple mode	118	1.857	1.048 to 3.288	0.036
Diastolic blood pressure	Weighted mode	118	1.813	1.027 to 3.202	0.043
Mean arterial pressure	MR Egger	83	2.134	0.888 to 5.128	0.094
Mean arterial pressure	Weighted median	83	2.317	1.783 to 3.011	<0.001
Mean arterial pressure	IVW	83	2.242	1.805 to 2.785	<0.001
Mean arterial pressure	Simple mode	83	2.650	1.336 to 5.256	0.007
Mean arterial pressure	Weighted mode	83	2.611	1.354 to 5.036	0.005
LDL cholesterol	MR Egger	143	1.747	1.239 to 2.463	0.002
LDL cholesterol	Weighted median	143	1.674	1.381 to 2.029	<0.001
LDL cholesterol	IVW	143	1.678	1.457 to 1.932	<0.001
LDL cholesterol	Simple mode	143	2.034	1.264 to 3.273	0.004
LDL cholesterol	Weighted mode	143	1.742	1.295 to 2.344	<0.001
HDL cholesterol	MR Egger	152	1.053	0.856 to 1.296	0.623
HDL cholesterol	Weighted median	152	0.878	0.749 to 1.028	0.106
HDL cholesterol	IVW	152	0.782	0.694 to 0.882	<0.001
HDL cholesterol	Simple mode	152	0.789	0.467 to 1.334	0.378
HDL cholesterol	Weighted mode	152	0.911	0.780 to 1.064	0.241
triglycerides	MR Egger	64	2.163	1.272 to 3.678	0.006
triglycerides	Weighted median	64	1.455	1.112 to 1.904	0.006
triglycerides	IVW	64	1.346	1.094 to 1.658	0.005
triglycerides	Simple mode	64	1.509	0.863 to 2.639	0.154
triglycerides	Weighted mode	64	1.469	0.992 to 2.176	0.059
Hypertension	MR Egger	12	1.624	1.082 to 2.437	0.041
Hypertension	Weighted median	12	1.216	1.061 to 1.394	0.005
Hypertension	IVW	12	1.319	1.152 to 1.509	<0.001
Hypertension	Simple mode	12	1.204	0.972 to 1.491	0.118
Hypertension	Weighted mode	12	1.212	1.041 to 1.412	0.031

MR: Mendelian randomization; LDL cholesterol: low-density lipoprotein cholesterol; HDL cholesterol: high-density lipoprotein cholesterol; SNP: single nucleotide polymorphisms; CI: confident interval.

**Table 5:** Results of multivariable analysis related to myocardial infarction

Exposures	Outcome	OR	95% CI	*P*-value
LDL cholesterol	Myocardial infarction	1.748	1.598 to 1.913	<0.001
HDL cholesterol	Myocardial infarction	0.807	0.721 to 0.904	<0.001
Diastolic blood pressure	Myocardial infarction	1.365	0.833 to 2.234	0.217
Systolic blood pressure	Myocardial infarction	0.781	0.474 to 1.285	0.330
Mean arterial pressure	Myocardial infarction	1.317	0.564 to 3.076	0.525
Hypertension	Myocardial infarction	1.355	1.189 to 1.543	<0.001
Triglycerides	Myocardial infarction	0.886	0.785 to 1.001	0.053

LDL cholesterol: low-density lipoprotein cholesterol; HDL cholesterol: high-density lipoprotein cholesterol; OR: odds ratio; CI: confident interval.

### Mediation MR analysis

**Figure 7:**
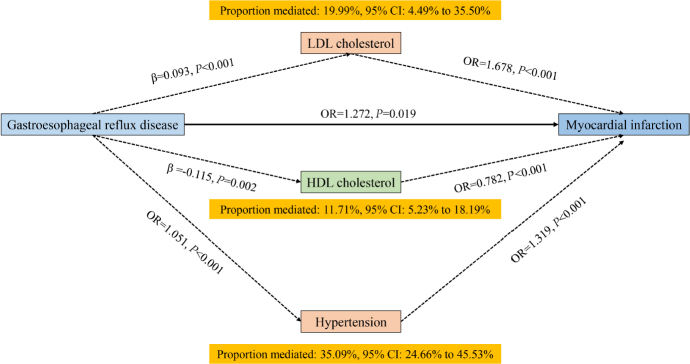
Mediation MR analysis. LDL cholesterol, HDL cholesterol and hypertension mediate the effects of gastroesophageal reflux disease on myocardial infarction. LDL cholesterol: low-density lipoprotein cholesterol; HDL cholesterol: high-density lipoprotein cholesterol.

We further employed mediation MR analysis to investigate how LDL cholesterol, HDL cholesterol, and hypertension mediate the effect of GERD on myocardial infarction. The results of the mediation MR analysis revealed that LDL cholesterol mediated 19.99% (95% CI: 4.49% to 35.50%) of the effect of GERD on myocardial infarction, HDL cholesterol mediated 11.71% (95% CI: 5.23% to 18.19%) of the effect of GERD on myocardial infarction, and hypertension mediated 35.09% (95% CI: 24.66% to 45.53%) of the effect of GERD on myocardial infarction. Detailed results are presented in Figure 7.

## Discussion

### Key finding

This MR study provides novel evidence supporting a causal relationship between GERD and significant alterations in blood pressure components, lipid profile, and an increased risk of cardiovascular diseases. Specifically, genetically predicted GERD exhibited positive associations with SBP, DBP, and MAP. Furthermore, our MR analyses revealed a substantial impact of GERD on lipid profile parameters, with genetically predicted GERD being associated with elevated levels of LDL cholesterol and triglycerides, while concurrently showing a negative impact on HDL cholesterol. Furthermore, our study revealed a significant association between genetically predicted GERD and a high risk of myocardial infarction and hypertension. Additionally, mediation MR analysis elucidated that LDL cholesterol (19.99% [95% CI: 4.49% to 35.50%]), HDL cholesterol (11.71% [95% CI: 5.23% to 18.19%]), and hypertension (35.09% [95% CI: 24.66% to 45.53%]) mediate the effect of GERD on myocardial infarction. Nevertheless, no noteworthy associations were detected between genetically predicted GERD and PP, total cholesterol, heart failure, and atrial fibrillation.

### GERD and blood pressure components

Presently, several prior studies have investigated the influence of GERD on blood pressure components. Ha *et al*.^[6]^ conducted a study involving 150 participants diagnosed with GERD and 292 non-GERD individuals, revealing no significant differences in SBP (129.2 ± 1.4 *vs*. 130.7 ± 1.0 mmHg, *P* = 0.390) and DBP (78.4 ± 0.9 *vs*. 77.6 ± 0.6 mmHg, *P* = 0.437) between GERD patients and those without GERD. Similarly, Kallel *et al*.^[7]^ included 54 participants diagnosed with GERD and 46 non-GERD participants, with no significant differences observed in SBP (122.07 ± 7.68 *vs*. 121.52 ± 12.9 mmHg, *P* = 0.16) and DBP (70.52 ± 12.04 *vs*. 70.23 ± 17.95 mmHg, *P* = 0.9) between GERD participants and those without GERD. Contrary to the studies performed by Ha^[6]^ and Kallel,^[7]^ Milovanovic *et al*.^[31]^ found that GERD patients had higher SBP and PP compared to age- and gender-matched participants. Furthermore, based on a large-scale study, Kim *et al*.^[32]^ found that GERD patients have higher SBP (121.0 [112.0–131.0 mmHg] ) compared to those without GERD (120.0 [110.0–130.0 mmHg]), while there was no significant difference in DBP between the two groups. It’s crucial to highlight that those previous studies had small sample sizes and couldn’t effectively account for confounding factors like age, gender, BMI, underlying diseases, or medication usage. In our study, utilizing MR analysis on a large-scale GWAS dataset, the presence of genetically predicted GERD significantly elevated SBP (β = 0.053, *P* = 0.036), DBP (β = 0.100, *P* < 0.001), and MAP (β = 0.106, *P* < 0.001), with no significant association observed with PP. The results of our study show different conclusions from previous research, likely attributed to the considerably larger sample size and the exclusion of potential confounding factors. For instance, participants with GERD are significantly older than non-GERD patients (44.59 ± 10.34 *vs*. 37.63 ± 14.41, *P* = 0.006) in Kallel’s study,^[7]^ while those with GERD have a significantly lower BMI than non-GERD individuals (23.9 ± 0.3 *vs*. 24.8 ± 0.2, *P* = 0.026) in Ha’s study.^[6]^ Thus, it is challenging to compare the differences in blood pressure components between GERD participants and non-GERD participants due to the potential influence of confounding factors. In our MR study, the influence of potential confounding factors has been eliminated, and the final conclusions are more accurate.

### GERD and lipid profile

The association between GERD and lipid profile remains uncertain. In Ha *et al*. study,^[6]^ a comparison of lipid profiles between GERD and non-GERD patients revealed a significant difference in triglycerides. GERD patients had significantly higher triglyceride levels than non-GERD counterparts (157.8 ± 19.5 *vs*. 120.5 ± 3.9 mg/dL, *P* = 0.013). However, there was no significant difference in total cholesterol and HDL cholesterol between GERD and non-GERD patients. Similarly, in Kallel *et al*. study,^[7]^ a comparison of lipid profiles between GERD and nonGERD patients did not identify significant differences in total cholesterol (106.33 ± 61.6 *vs*. 90 ± 37.61 mg/dL, *P* = 0.14) and HDL cholesterol (47.38 ± 12.12 *vs*. 52.41 ± 22.02 mg/dL, *P* = 0.16). Additionally, in Kim *et al*. study,^[32]^ the comparison of lipid profiles between GERD and non-GERD patients revealed lower LDL cholesterol and HDL cholesterol in GERD patients, along with higher total cholesterol and triglyceride levels compared to those without GERD. In contrast to prior studies, this MR study revealed noteworthy differences in lipid profiles between GERD and non-GERD patients. Specifically, genetically predicted GERD demonstrated a significant association with elevated LDL cholesterol (β = 0.093, *P* < 0.001) and triglycerides (β = 0.153, *P* < 0.001), alongside an association with reduced HDL cholesterol (β = -0.115, *P* = 0.002). However, no significant association was noticed between genetically predicted GERD and total cholesterol. The considerable sample size (> 200,000 individuals) and careful control for confounding factors in this MR study likely contribute to more stable results compared to previous research, as outlined above.

### GERD and cardiovascular diseases

Nowadays, there are limited studies systematically assessing the impact of GERD on cardiovascular diseases. In a nationwide population-based study, Lei *et al*.^[33]^ enrolled 54, 422 patients diagnosed with GERD and 269,572 age- and gender-matched controls. After a mean follow-up of 3.3 years, they identified a notable association between GERD and a high risk of myocardial infarction (hazard ratio [HR] = 1.48, 95% CI: 1.31 to 1.66, *P* < 0.001). In another large sample study, Eisa *et al*.^[34]^ similarly noted an increased risk of myocardial infarction linked to the presence of GERD, and this association remained exist after adjusting for potential confounding factors. Aligning with prior research, our MR study also revealed a substantial association between genetically predicted GERD and an elevated risk of myocardial infarction (OR = 1.272, 95% CI: 1.040 to 1.557, *P* = 0.019). Presently, there is a lack of research directly evaluating the causative relationship between GERD and hypertension. In a study conducted by Li *et al*.,^[35]^ 86 hypertensive patients were enrolled, revealing that 44.2% of those with hypertension also experienced GERD, indicating a notable association between hypertension and GERD. Another study ^[36]^ found that in hypertensive patients, the prevalence of silent GERD and GERD were 15.1% and 31.4%, respectively. However, while those studies suggest an association between GERD and hypertension, the potential causative relationship remains unclear. To our knowledge, the findings of our MR study are the first to reveal that genetically predicted GERD is associated with an increased risk of hypertension (OR = 1.357, 95% CI: 1.222 to 1.507, *P* < 0.001), systematically elucidating the causal relationship between GERD and hypertension.

This study performed by Sun *et al*.^[37]^ is the initial investigation into the association between GERD and heart failure, and found that the presence of GERD is associated with a higher risk of heart failure. However, it needs to be emphasized that the study by Sun *et al*.^[37]^ did not eliminate instrumental variables significantly associated with confounding factors, thereby leading to potential bias in the final conclusion. Contrary to the study by Sun *et al*.^[37]^ our findings indicate that genetically predicted GERD is not significantly associated with a high risk of heart failure. One major reason attributing to the difference is that instrumental variables associated with confounding factors, such as age, gender, BMI, obesity, smoking, alcohol consumption, waist circumference, hip circumference, waist-hip ratio, depression, anxiety, HbA1c levels, blood glucose, tumors, diabetes, stress, obstructive sleep apnea, chronic kidney disease, multiple sclerosis, rheumatoid arthritis, potential diseases, and medication usage, were excluded from our study, whereas the exclusion of confounding factors is not mentioned in Sun’s study.^[37]^ In that case, the conclusions of our study are more accurate because the influence of confounding factors on the findings can be considered negligible.

On the other hand, there is significant controversy regarding the causal relationship between GERD and atrial fibrillation. A study^[8]^ with 29,688 diagnosed GERD patients and 29,597 non-GERD patients suggested that GERD independently contributes to the occurrence of atrial fibrillation (HR = 1.31, 95% CI: 1.06 to 1.61, *P* = 0.013). Another study provided evidence that the presence of GERD increases the risk of atrial fibrillation in individuals below the age of 60.^[9]^ Similarly, Sun *et al*. study also found that the presence of GERD also significantly increased the risk of atrial fibrillation. In contrast, Bunch *et al*. study^[10]^ found no association between the presence of any GERD and the risk of atrial fibrillation, and even the frequency of GERD was unrelated to the risk of atrial fibrillation. The results of this MR study align with Bunch *et al*. findings,^[10]^ indicating that genetically predicted GERD is not associated with the risk of atrial fibrillation after eliminating the potential influence of confounding factors. Considering that the impact of confounding factors on the outcome was eliminated in this MR study, the conclusion is more accurate and reliable.

Additionally, this study has, for the first time, found that LDL cholesterol mediated 19.99% (95% CI: 4.49% to 35.50%), HDL cholesterol mediated 11.71% (95% CI: 5.23% to 18.19%), and hypertension mediated 35.09% (95% CI: 24.66% to 45.53%) of the effect of GERD on myocardial infarction, which has not been reported in previous research. This provides important evidence for reducing the risk of myocardial infarction in GERD patients, indicating that lowering LDL cholesterol, controlling hypertension, and increasing HDL cholesterol can help reduce the risk of myocardial infarction in GERD patients. Furthermore, this evidence potentially suggests that lipid-lowering and antihypertensive medications may significantly reduce the risk of myocardial infarction in GERD patients. However, this hypothesis still requires further evidence for clarification.

### Potential mechanism

The observed causal relationship between GERD and alterations in blood pressure components, lipid profile, and increased risk of cardiovascular diseases suggests potential underlying mechanisms that warrant exploration. While the precise pathways remain to be fully elucidated, several hypotheses can be considered. GERD is known to trigger local and systemic inflammation.^[38,39]^ Chronic inflammation caused by the presence of GERD may contribute to endothelial dysfunction,^[40]^ atherosclerosis,^[33]^ and subsequent cardiovascular events. Inflammatory mediators could potentially influence blood pressure regulation, lipid metabolism^[41]^ and ultimately led to the occurrence of cardiovascular diseases. In addition, GERD-related esophageal irritation might damage cardiac autonomic dysfunction. Prior research suggests a disruption in the autonomic nervous system among individuals GERD, particularly affecting the sympathetic and parasympathetic components.^[31]^ However, it seems that compromised parasympathetic nervous system function aligns more closely with the onset and development of GERD. Furthermore, GERD-associated alterations in lipid profile parameters could be linked to metabolic disturbances.^[7,42]^ This may involve disruptions in lipid metabolism pathways, leading to increased levels of LDL cholesterol and triglycerides, and decreased levels of HDL cholesterol.

### Clinical implication

The results of our MR study, elucidating the potential causal association between GERD and blood pressure components, lipid profile, and cardiovascular diseases, have substantial clinical implications. Firstly, healthcare providers should consider integrating cardiovascular risk assessments into the care plans for GERD patients. Regular monitoring of blood pressure components and lipid profiles may be necessary to identify individuals at a high risk of cardiovascular diseases. Secondly, the identified associations underscore the necessity for tailoring treatment strategies for GERD patients. Customizing interventions based on an individual’s cardiovascular risk profile, taking into account factors such as blood pressure and lipid profile, might improve overall treatment outcomes. Thirdly, with the potential causal association between GERD and cardiovascular diseases in mind, early intervention in GERD management may hold broader implications for cardiovascular health. Thus, addressing GERD comprehensively could play a role in mitigating cardiovascular risks. Finally, this MR study emphasizes the importance for research into therapeutic targets that simultaneously address both GERD symptoms and potential cardiovascular complications. Therefore, investigating medications or lifestyle interventions targeting both conditions could provide more holistic and effective treatment strategies.

Our study suggests that GERD is associated with alterations in blood pressure components, lipid profile parameters, and cardiovascular diseases, which could inform future guidelines and interventions aimed at improving patient outcomes. However, it is crucial to note that further research is needed to validate these findings and expand our understanding of the mechanisms underlying the observed relationships between GERD and cardiovascular/ metabolic complications. Firstly, longitudinal studies are necessary to further establish causal relationships between GERD and cardiovascular/metabolic outcomes in large sample size populations. Secondly, further research should focus on the biological mechanisms that link GERD to changes in blood pressure, lipid profiles, and cardiovascular diseases. This could involve exploring genetic, molecular, and physiological pathways.

### Limitations

Several limitations need to be acknowledged. Firstly, we assume that our chosen genetic instruments adequately represent the entire spectrum of factors related to GERD. Considering the multifaceted nature of GERD with various environmental and genetic contributors, any gaps or inaccuracies in our representation may introduce bias into the estimates. Secondly, while MR is adept at establishing causal inference, it falls short in revealing the specific mechanisms underlying these associations. Further research is crucial to unravel the biological pathways linking GERD to blood pressure, lipid metabolism, and cardiovascular diseases. Thirdly, while we selected SNPs robustly associated with GERD from GWAS, there may still be unaccounted genetic variants influencing the outcomes through pathways other than GERD. Additionally, our findings may be affected by population characteristics of the GWAS data used. Lastly, our study concentrated on common genetic variants, potentially overlooking rare variants or gene-environment interactions that might influence the observed associations.

## Conclusion

In summary, our MR study strongly suggests a potential cause and effect association between GERD and substantial alterations in blood pressure components, lipid profile, and an elevated risk of cardiovascular diseases. The identified links between genetically predicted GERD and increased SBP, DBP, MAP, LDL cholesterol, and triglycerides, along with a decreased level of HDL cholesterol, as well as a high risk of myocardial infarction and hypertension, highlight the complicated associations between GERD and cardiovascular health. Furthermore, this study has further discovered that LDL cholesterol, HDL cholesterol, and hypertension mediate the effect of GERD on myocardial infarction, providing important evidence for reducing the risk of myocardial infarction in GERD patients. These findings have significant clinical implications, emphasizing the need for cardiovascular risk assessment and personalized treatment strategies for individuals with GERD. However, further investigation is necessary to demonstrate the underlying mechanisms and confirm these associations across diverse populations.
